# Impact of genetic test interpretation on a *VPS13B* missense variant in Cohen syndrome

**DOI:** 10.3389/fnins.2024.1488133

**Published:** 2024-12-11

**Authors:** Gudrun Schottmann, Carmen Martínez Almudéver, Julia C. M. Knop, Eun Kyung Suk, Zianka Meyer, Jürgen Kohlhase, Nastassja Himmelreich, Jirko Kühnisch, Claus-Eric Ott, Wenke Seifert

**Affiliations:** ^1^Zentrum für Sozial-und Neuropädiatrie (DBZ), Vivantes Klinikum Neukölln, Berlin, Germany; ^2^Institute of Cell Biology and Neurobiology, Charité - Universitätsmedizin Berlin, corporate member of Freie Universität Berlin und Humboldt-Universität zu Berlin, Berlin, Germany; ^3^Praxis für Humangenetik, Berlin, Germany; ^4^Diagenom GmbH, Rostock, Germany; ^5^Zentrum für Humangenetik, SYNLAB MVZ Humangenetik Freiburg, Tübingen, Germany; ^6^Zentrum für Humangenetik Tübingen, Tübingen, Germany; ^7^CeGaT, Tübingen, Germany; ^8^Experimental and Clinical Research Center (ECRC), a cooperation been the Max Delbrück Center for Molecular Medicine in the Helmholtz Association and Charité—Universitätsmedizin Berlin, Berlin, Germany; ^9^Institute of Physiology, Brandenburg Medical School (MHB) Theodor Fontane, Brandenburg an der Havel, Germany; ^10^Institute for Medical Genetics and Human Genetics, Charité—Universitätsmedizin Berlin, corporate member of Freie Universität Berlin und Humboldt Universität zu Berlin, Berlin, Germany

**Keywords:** VPS13B, Cohen syndrome, missense variant, functional testing, Golgi complex

## Abstract

**Introduction:**

Cohen syndrome (CS) is an early-onset pediatric neurodevelopmental disorder characterized by postnatal microcephaly and intellectual disability. An accurate diagnosis for individuals with CS is crucial, particularly for their caretakers and future prospects. CS is predominantly caused by rare homozygous or compound heterozygous pathogenic variants in the vacuolar protein sorting-associated 13B (*VPS13B*) gene, which disrupt protein translation and lead to a loss of function (LoF) of the encoded VPS13B protein.

**Methods:**

The widespread incorporation of next-generation sequencing approaches in genetic diagnostics increases the number of individuals carrying *VPS13B* mutant alleles. At the same time, it increases the detection of variants of unknown clinical significance, necessitating further functional pathogenicity validation.

**Results:**

In this study, we present a family with two CS patients. Within this family, four rare *VPS13B* variants were detected: c.710G > C, p.Arg237Pro; c.6804delT, p.Phe2268Leufs*24; c.7304C > T, p.Ala2435Val; and c.10302T > A, p.Tyr3434*. These variants challenge the interpretation of their disease-causing role. Specifically, the variants c.6804delT, p.Phe2268Leufs*24 and c.710G > C, p.Arg237Pro were detected in trans configuration and are considered to be causing CS genetically. The functional characterization of the missense variant c.710G > C, p.Arg237Pro shows diminished localization at the Golgi complex, highlighting its clinical relevance and supporting its classification by the American College of Medical Genetics and Genomics (ACMG) as likely pathogenic, class 4.

**Discussion:**

Overall, we emphasize the need for combining genetic and functional testing of VPS13B missense variants to ensure accurate molecular diagnosis and personalized medical care for CS patients.

## Introduction

In recent decades, human genetics has enabled the rapid and comprehensive identification of genetic variants and their association with neurological diseases (Satam et al., 2023). However, we emphasize the importance of systematic phenotyping and functional studies, especially when the interpretation of variants of unknown significance (VUS) leaves uncertainty regarding their pathogenic character.

In this study, we present two brothers with the phenotype of Cohen syndrome (CS, MIM: #216550). CS is a rare autosomal recessive genetic condition that is primarily characterized by developmental delay, postnatal microcephaly, and distinctive facial gestalt with wave-shaped eyelids and a short philtrum (Kolehmainen et al., 2003; El Chehadeh-Djebbar et al., 2013). The clinical diagnosis of CS is facilitated by the presence of progressive retinal dystrophy and/or neutropenia. Additional facultative clinical signs include myopia, childhood hypotonia, joint laxity, a cheerful disposition, and autism spectrum disorder (ASD)-like behavior (Katzaki et al., 2007; Gueneau et al., 2014). Genetically, CS is predominantly caused by splice site mutations, frameshift indels, or nonsense variants, which typically results in a complete loss of function (LoF) of the encoded vacuolar protein sorting-associated 13B (VPS13B) (Seifert et al., 2009). VPS13B belongs to the VPS13 family of bridge-like lipid transfer proteins, suggesting that it facilitates lipid transport between adjacent membranes (Vacca et al., 2024; Mochida et al., 2004; Hanna et al., 2023). At the cellular level, VPS13B is primarily localized to the Golgi complex. It plays a crucial role in preserving the morphology and function of the Golgi, potentially influencing membrane organization and intracellular membrane transport (Vacca et al., 2024; Seifert et al., 2011). Recent studies using neuronal models, including iPSC-derived neurons and animal models of CS, have indicated that VPS13B depletion is linked to neurodevelopmental abnormalities, such as axonal elongation defects, defective synaptogenesis, and increased autophagic flux (Seifert et al., 2015; Lee et al., 2020; Lee et al., 2020). However, a clear genotype–phenotype correlation or functional correlation regarding the differences in the clinical expression of CS has not yet been established.

In contrast, the interpretation of *VPS13B* missense variants is challenging due to the lack of biochemical and pathomechanistic characterization of most VPS13B protein regions. Although *in silico* analysis supports predictions of pathogenicity, functional evidence provides much stronger validation for the pathogenic character of a genetic variant. To overcome this limitation, we established a cellular detection assay that measures the subcellular distribution of the encoded VPS13B protein in the Golgi apparatus and the cytosol after immunostaining (Zorn et al., 2022). In this study, we employed a quantitative subcellular analysis of VPS13B mutant protein distribution to classify the familial *VPS13B* missense VUS, thereby validating the genetic diagnosis for CS.

## Methods

### Family consent

Written informed consent for genetic testing and the use of data for publication was obtained from each participant. Parental consent was secured for both siblings under 18 years of age.

### Genetic testing

DNA was isolated from peripheral EDTA blood. Both patients underwent initial Sanger sequencing of the entire coding region of the *VPS13B* gene (exon 2–62, including 20 bp of the 3′ and 5′ intron regions) using standard procedures with a 3130XL Genetic Analyzer (Applied Biosystems). All details can be obtained upon request. Quadro analysis of the *VPS13B* gene using NGS-based whole exome sequencing was performed on both patients and their parents to reevaluate the variants, technically confirm allelic distribution, and ascertain the *de novo* status. Enrichment of the coding regions, adjacent intronic regions, and other non-coding, disease-relevant regions of the *VPS13B* candidate region was performed using *in-solution* hybridization. For exome capture, we used enrichment kits from Twist Bioscience. High-throughput sequencing was conducted using the Illumina NovaSeq 6,000/NovaSeq X Plus System. Adapter sequences were removed using Skewer, a tool designed for trimming high-throughput sequencing reads. The resulting sequences were aligned to the human reference genome (hg19) using the Burrows–Wheeler Aligner sequences. The sequences that were not uniquely aligned or were identified as duplicates, most likely due to PCR amplification, were removed to ensure data accuracy. Sequencing data were processed using Illumina bcl2fastq2 software, which converts raw BCL files generated by the sequencer into FASTQ files for further analysis. The remaining high-quality sequences were annotated for sequence variants using various internal and external databases, including the Illumina database and the VPS13B MANE PLUS CLINICAL transcript ENST00000358544, NM_017890.5, comprising 12,069 bp. However, in this study, all variants were aligned to the ubiquitously expressed and conserved variant *VPS13B* ENST00000357162.7, NM_152564.5, comprising 11,994 bp and encoding 3,997 amino acids (aa) as the reference.

### Reagents and antibodies

All reagents were obtained from Sigma-Aldrich, Roth or Merck, unless stated otherwise. The following commercial antibody was used: Mouse anti-GM130 (BD Transductions Laboratories, Cat. No. 610822). Rabbit anti-VPS13B (442) was described earlier (Seifert et al., 2011). Secondary antibodies for immunofluorescence included donkey anti-mouse-Cy3, anti-rabbit-Cy3, and anti-rabbit-Cy2 (all from Dianova GmbH). 6-Diamidino-2-phenylindole (DAPI) (Invitrogen) was used for nuclear DNA staining.

### Cloning of the *VPS13B* variant expression vectors by site-directed mutagenesis

Missense variants were annotated using the *VPS13B* transcript [Ensembl, ENST00000357162.6 (VPS13B-202); RefSeq, NM_152564.5]. For transient expression, the full-length human *VPS13B* construct encoding the VPS13B missense variant p.Arg237Pro (pcDNA3.1_VPS13B-mutR237P) was cloned using site-directed mutagenesis PCR (Reikofski and Tao, 1992). Briefly, the full-length wild-type human construct pcDNA3.1_VPS13B-wt (Seifert et al., 2011) was amplified using primer pairs containing the corresponding missense variants in VPS13B using PrimeSTAR GXL DNA Polymerase (NEB Inc.) according to the manufacturer’s instructions. The PCR products were subsequently treated with the KLD enzyme mix (NEB Inc.), which eliminated the methylated pcDNA3.1_VPS13B-wt vectors and prepared them for the DNA ligation reaction. The integrity and correct mutagenesis of all cloned vectors were confirmed through Sanger sequencing.

### Cell culture and transfection

HeLa-cells (ATCC) were cultured at 37°C in a 5% CO_2_ atmosphere using DMEM culture medium supplemented with 5% FCS and 1% glutamine. Transient transfection of the expression vectors was performed at approximately 50% confluence using Lipofectamine (Invitrogen, Thermo Fisher) according to the manufacturer’s instructions. The variant of interest was pcDNA3.1_VPS13B-mutArg237Pro, while pcDNA3.1_VPS13B-mutAla590Thr and pcDNA3.1_hVPS13B-mutGlu2704Arg served as negative and positive controls, respectively. Cell harvesting for further analysis was performed 24–48 h post-transfection.

### Immunofluorescence

For visualization of intracellular protein expression and localization, the cells were cultured on coverslips and, if required, transfected (see above). The cells on the coverslips were fixed using 3% (w/v) paraformaldehyde in 1x PBS at 4°C and permeabilized in 0.5% (v/v) Triton X-100 and 1% (w/v) BSA in 1x PBS. Primary antibodies were incubated in 1% (w/v) BSA diluted in 1x PBS for at least 8 h at 4°C. After rinsing with PBS, secondary antibodies were incubated in 1% (w/v) BSA diluted in 1x PBS for 2 h at 4°C. Subsequently, the coverslips were attached to slides using ImmuMount (ThermoFisher). The visualization was performed using a spinning disk microscope (Zeiss), with a comparable cellular imaging level and identical imaging conditions.

### ImageJ and statistical analysis

Image analysis and Golgi enrichment were performed using ImageJ. Two different regions of interest (ROIs) were defined for each cell separately, as previously described (Zorn et al., 2022). Briefly, one ROI outlining the cell border measured the total VPS13B immunofluorescence of the cell (total cell ROI), while the second ROI outlining the GM130-positive Golgi structure (Golgi ROI) measured the Golgi-associated immunofluorescence of VPS13B. The percentage of Golgi-associated VPS13B fluorescence compared to the total VPS13 cell fluorescence intensity was calculated. The graphical representation and statistical analysis were carried out using GraphPad Prism software. The VPS13B variants pcDNA3.1_VPS13B-mutAla590Thr and pcDNA3.1_hVPS13B-mutGlu2704Arg were previously described (Zorn et al., 2022) and included in the study as Golgi enrichment-affecting negative and positive controls for the VPS13B missense variant, respectively.

### *In silico* prediction and variant classification for the evaluation of pathogenicity

All variant pathogenicity prediction algorithms and the ACMG classification (ACMG/ACGS 2020 guidelines, version 4.01) were conducted as previously described (Zorn et al., 2022). Truncating *VPS13B* variants were classified as decribed (Abou Tayoun et al., 2018).

## Results

### Patients and family

Here, we report the phenotype of two brothers with clinical manifestations indicating CS.

Patient 1 was born at term (41 weeks) via spontaneous delivery, with a relatively low birth weight of 3,030 g (P16, −1.01 SD). He exhibited respiratory adaptation disorder and muscular hypotonia. A cranial MRI scan at birth revealed a structurally unremarkable brain with slightly enlarged cerebrospinal fluid spaces. After birth, the patient developed postnatal microcephaly, with a head circumference of 41 cm at the age of 1 (<P1, −5.19 SD), and a psychomotor developmental delay. All developmental milestones were met at delayed time points. The patient was able to sit independently at the age of 1.5 years, walked independently by the age of 4, and has remained non-verbal to date. Myopia was diagnosed at the age of 4. He is usually in a friendly mood and shows limited understanding of simple content. Moreover, within the first year, the patient was noted to have recurrent bronchial infections and neutropenia. Other symptoms included feeding difficulties, small genitalia, and bilateral undescended testis.

Patient 2, the younger brother, was born via spontaneous delivery at term after a pregnancy complicated by maternal diabetes requiring insulin therapy. At 6 months of age, developmental delay, muscular hypotonia, and feeding difficulties became evident. He developed distinct postnatal microcephaly and, despite intensive supportive therapy, showed slow developmental progress. He was able to sit unaided at 21 months and has been walking with support since the age of 3 years. Sleeping disorders and feeding difficulties have been ongoing to this day. The boy frequently shows high irritability and exhibits autistic features, including reduced eye contact, stereotypical movements, and social withdrawal. His mother reports that he often appears to be in his own world and lacks a recognizable need for social contact, which is in line with previous reports of autistic features in CS (Table 1).

**Table 1:** Summary of the clinical characteristics of both related brothers with CS.

CS features	Patient 1	Patient 2
Age at clinical assessment	8 y	3 y 3 m
Facial dysmorphism	+	+
Postnatal microcephaly	+	+
Growth chart		
At birth: gestational age/weight/length/head circumference	41 w/3030 g/n.s./n.s.	40 w/3130 g/48 cm/34 cm
Actual: age/weight/length/head circumference	8 y/26.2 kg/121.5 cm/46.7 cm	3 y/10.2 kg/85 cm/43.9 cm
Developmental delay	+	+
Head control	Unknown	4 m
Sitting unsupported	18 m	21 m
Walking unsupported	4 y	Supported walking at 3y
Speaking words	Non-verbal	Non-verbal
Autistic features	−	+
Myopia/retinopathy	+/−	−/−
Neutropenia	+	−
Congenital muscular hypotonia	+	+
Other	Recurrent infections, feeding difficulties until age 4 (improving since the age of 4), bilateral nondescensus testis, and small genitalia	Cranial flattening right >left, constant feeding difficulties, and sleeping difficulties

+ present, − absent, and n.s., not specified.

### Sequencing results

**Figure 1:**
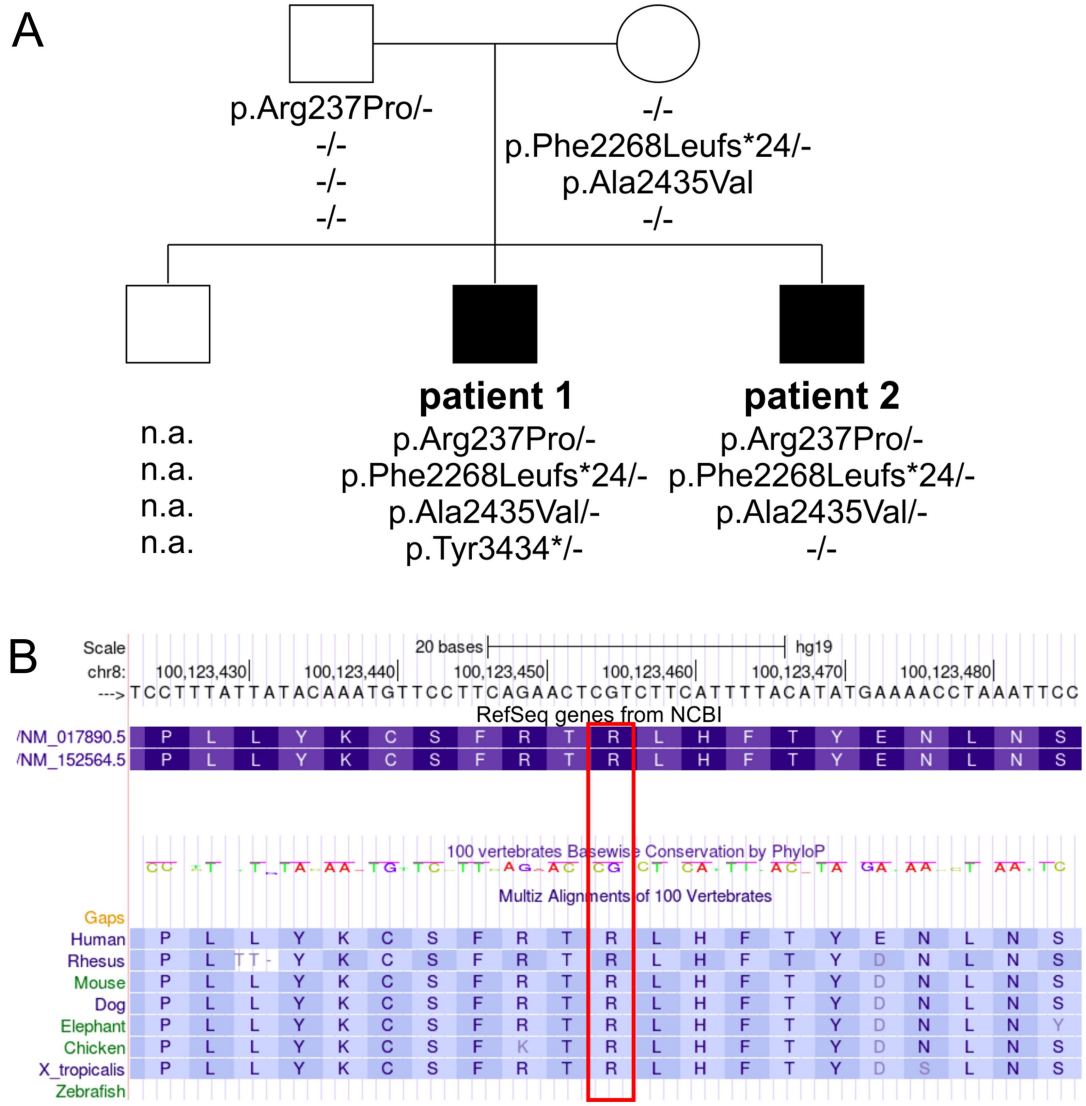
Pedigree and genetic findings in the family with CS. (A) Squares and circles symbolize males and females, respectively. Filled symbols indicate affected individuals, while unfilled symbols represent healthy individuals. The *VPS13B* genotypes are depicted below each symbol (n.a., no DNA available). (B) A partial screenshot of the *VPS13B* gene region on chr8:100,123,423–100,123,486 from the UCSC genome browser shows the conservation plot of the VPS13B p.Arg237 variant (highlighted in a red box), with conservation extending down to *Xenopus tropicalis*.

Chromosomal analysis and microarray comparative genomic hybridization (CGH) were unremarkable, indicating no major chromosomal abnormalities. Initial Sanger sequencing of the *VPS13B* gene was performed based on the clinically distinct phenotype of CS in Patient 1. Further molecular testing of both patients and the parents was performed using candidate region *in-solution* hybridization enrichment and high-throughput sequence analysis using the Illumina NovaSeq 6000/NovaSeq X Plus System. Next-generation sequencing-based copy number variations were calculated based on the sequences that could be assigned to a genomic position using an internally developed method based on sequencing depth. No genomic copy number imbalances were detected that could explain the patients’ phenotype. Further annotation of the high-throughput sequencing data using various internal and external databases for the sequence variants in the candidate region of *VPS13B* confirmed two pathogenic heterozygous variants and two heterozygous VUS in the older patient (Patient 1): c.6804delT, p.Phe2268fs*24 (Rafiq et al., 2015); c.10302T > A, p.Tyr3434*; c.710G > C, p. Arg237Pro; and c.7304C > T, p.Ala2435Val. In the younger patient (Patient 2), sequencing of the candidate region of *VPS13B* identified one pathogenic heterozygous variant and two heterozygous VUS: c.6804delT, p.Phe2268fs*24 (Rafiq et al., 2015); c.710G > C, p. Arg237Pro; and c.7304C > T, p.Ala2435Val. Subsequent analysis of the parents suggested that the mother carried the pathogenic variant c.6804delT, p.Phe2268fs*24 and the VUS c.7304C > T, p.Ala2435Val, which were inherited by both brothers. The second pathogenic variant, c.10302T > A, p.Tyr3434*, in Patient 1, was not inherited from the mother or the father, suggesting a *de novo* agent, germ cell mosaicism, or a somatic event. The father carried the heterozygous *VPS13B* VUS c.710G > C, p. Arg237Pro, which was inherited by both brothers (Figure 1; Table 2).

**Table 2:** Variant pathogenicity interpretation was conducted according to the ACMG standards and guidelines (Richards et al., 2015).

Variant (Protein change)	Arg237Pro	Phe2268Leufs*24	Ala2435Val	Tyr3434*
Population data				
Database (dbSNP)	n.d.	n.d.	rs752558975	n.d.
gnomAD allele frequency	n.d.	n.d.	0.000003098	n.d.
gnomAD allele count	0 Arg237Cys: 26/1601682 Arg237His: 18/1601318	0	5/1614036	0
gnomAD number of homozygotes	0 Arg237Cys: 0 Arg237His: 0	0	0	0
Computational and predictive *in silico* algorithms				
Character	Missense	Frameshift	Missense	stop
Grantham score	103	n.a.	64	n.a.
Mutation taster (*p*-value)	0.999999996377372	1 (NMD)	0.999999927453442	1 (NMD)
PolyPhen-2 (HumVar)	0.997 (pathogenic)	n.a.	0.997 (pathogenic)	n.a.
MutPred 2 probability	0.914 (pathogenic)	n.a.	0.627 (likely pathogenic)	n.a.
SNPs&GO—PhD-SNP	0.940 (disease)	n.a.	0.132 (neutral)	n.a.
SNPs&GO—SNPs&GO	0.769 (disease)	n.a.	0.030 (neutral)	n.a.
SIFT Score	0.00 (intolerant)	n.a.	0.00 (intolerant)	n.a.
Affected PROSITE and ELM Motifs	ELME000012, ELME000063, ELME000136, ELME000137, ELME000159, ELME000358	Truncation/NMD, LoF expected	ELME000052, ELME000159, ELME000358	Truncation/NMD, LoF expected
Functional data—*in vitro* data (cell culture)				
Golgi localization	Decreased	Not tested	Not tested	Not tested
Segregation data				
Co-segregation within the family (autosomal recessive)	Yes	Yes	No	No
Allelic data				
	Paternal allele	Maternal allele	Maternal allele	Paternal allele (*de novo*)
Literature				
	Unpublished	Rafiq et al. (2015)	Unpublished	Unpublished
ACMG categories				
**Population data**	**PM2**	**PM2**	**PM2**	**PM2**
**Predictive data**	**PP3**	**PVS1**	**Inconsistent**	**PVS1**
**Functional data**	**PS3**	**n.a.**	**n.a.**	**n.a.**
**Segregation**	**PP1**	**PP1**	**n.a.**	**n.a.**
**De novo data**	**n.a.**	**n.a.**	**n.a.**	**PS2**
**Allelic data**	**PM3**	**PM3**	**BP2**	**n.a. (ambiguous for familiar CS diagnosis)**
**Reinterpretation according to the ACMG**	**Likely pathogenic (class 4)**	**Pathogenic (class 5)**	**Low VUS (class 3)**	**Pathogenic (class 5)**

**Figure 2:**
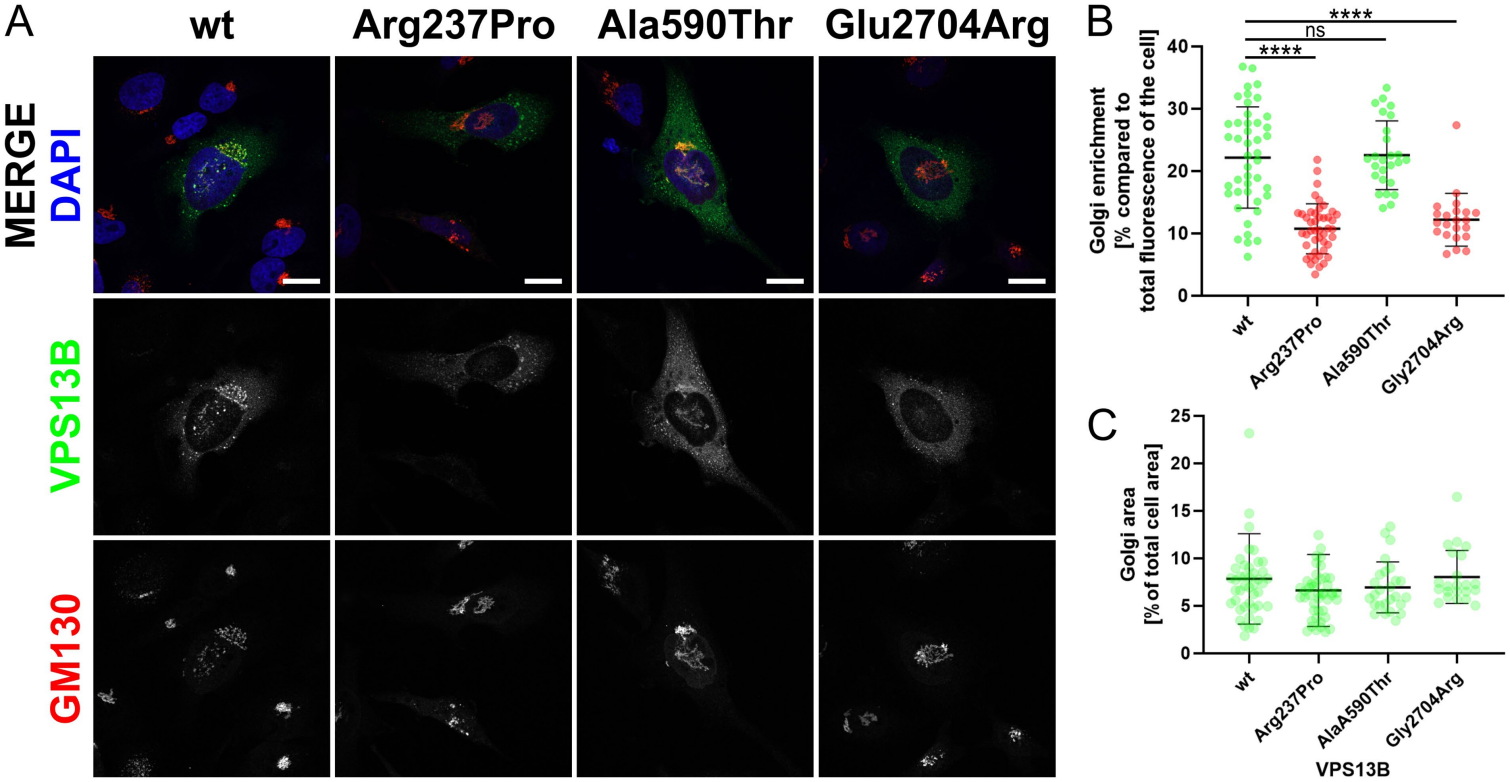
Golgi localization. (A) *HeLa* cells were transfected with different pcDNA3.1_VPS13B constructs, encoding either wild-type (wt) or mutated VPS13B (missense variants p.Arg237Pro, p.Ala590Thr, p.Glu2704Arg). Twenty-four hours post-transfection, all cells were processed for immunostaining of VPS13B (green) and GM130 (red). Imaging was performed using a Zeiss spinning disk microscope. Nuclei were stained with DAPI (blue). The scale bar represents 20 μm. (B) Quantitative analysis of the subcellular Golgi enrichment of VPS13B was conducted using ImageJ. Two ROIs were defined for each analyzed cell: one total cell ROI, drawn around the cell area, and one Golgi ROI, drawn around the GM130-positive Golgi area. Statistical analysis was conducted on the Golgi enrichment by calculating the percentage of the VPS13B fluorescence intensity at the Golgi compared to the total cellular fluorescence intensity. (C) Analysis of the Golgi area of VPS13B was conducted using ImageJ. Both ROIs were used to measure the area. Statistical analysis was performed by calculating the percentage of the occupied Golgi area compared to the total cellular area.

For population data, the gnomAD database was used (Chen et al., 2024). To obtain computational and predictive *in silico* data, we applied analyses using different tools: Grantham (Grantham, 1974), MutationTaster (Schwarz et al., 2014), PolyPhen-2 (Adzhubei et al., 2013), SNP&GO (Calabrese et al., 2009), MutPred2 (Pejaver et al., 2020), and the SIFT Score (Kumar et al., 2009). Functional data for p.Arg237Pro were obtained using *in vitro* analyses in HeLa cells (refer to Figure 2). Allelic data were obtained from the pedigree and the segregation of the CS phenotype, along with the previously identified genotype of the individuals within the family. The literature was searched for previously published genotypes. The reinterpretation of all variants was performed according to the ACMG standards and guidelines (Deignan et al., 2019). n.a., not applicable and n.d., not documented.

### Variant pathogenicity prediction algorithms

To understand the complex genetic and clinical data, we interpreted the *VPS13B* variants according to the ACMG standards and guidelines (Deignan et al., 2019). The Phe2268Leufs*24 variant has been previously reported to cause CS (Table 2, ClinVarID: 817631) (Rafiq et al., 2015; Zare Ashrafi et al., 2023). The p.Tyr3434* variant was detected in Patient 1, and it is classified as a pathogenic variant due to its deleterious nature. However, this variant did not co-segregate with the CS phenotype within the family, likely indicating a *de novo* mutation, germ cell mosaicism, or a somatic event. Paternity and maternity for both patients were confirmed. The missense variants were further processed using standard computational and predictive *in silico* algorithms. In this study, the major difference between both missense variants was predicted using SNPs&GO analyses (Calabrese et al., 2009), which indicated a disease-causing prediction for p.Arg237Pro and a neutral character for p.Ala2435Val (Table 2). The disease-causing prediction of pArg237Pro was further supported by its conservation down *to Xenopus tropicalis* (Figure 1B), while p.Ala2435Val was excluded as a cause of the CS phenotype due to its co-segregation as a complex maternal allele with p.Phe2268Leufs*24. Overall, the segregation of p.Arg237Pro with the CS phenotype suggested that this missense variant caused autosomal recessive CS in both affected brothers, along with the p.Phe2268Leufs*24 variant.

### Golgi localization

To date, the analysis of cellular VPS13B distribution is the only simple and reliable method to investigate the effects of *VPS13B* missense variants (Zorn et al., 2022). This method involves cloning the respective missense variant into a mammalian expression vector (pcDNA3.1_VPS13B) and subsequent overexpression in standard cell cultures, such as HeLa cells (Zorn et al., 2022). Using this approach, we showed that the wild-type VPS13B protein localizes to the Golgi complex, as indicated by its co-localization with the Golgi matrix protein GM130 (Figure 2A). As controls, we analyzed two previously reported *VPS13B* missense variants regarding their subcellular localization (Zorn et al., 2022). The p.Ala590Thr variant exhibited a subcellular distribution similar to the wild-type VPS13B protein and was therefore used as a negative control. In contrast, the p.Gly2704Arg variant showed reduced localization to the Golgi complex and was used as a positive control, indicating a disruption in subcellular targeting. The analysis of the p.Arg237Pro variant revealed a significant reduction in Golgi enrichment of the mutated VPS13B protein (Figures 2A,B). It is important to note that the overall area covered by the Golgi complex was not affected; therefore, all variants are comparable to the wild-type VPS13B protein in this respect (Figure 2C).

## Discussion

CS is a rare neurodevelopmental autosomal recessive condition mainly characterized by developmental delay and postnatal microcephaly. In this study, we present a family with two affected brothers who exhibit the obligate clinical CS signs, such as postnatal microcephaly, developmental delay, and distinctive facial gestalt. When we compared the two brothers, we found divergent clinical signs: for example, certain autistic features were exhibited by Patient 2 whereas myopia and neutropenia were detected in Patient 1 only. Genetic testing of *VPS13B* in the family revealed two LoF variants, p.Phe2268fs*and p.Tyr3434*, as well as two missense variants, p.Arg237Pro and p.Ala2435Val. The heterozygous variant p.Phe2268fs*24 was found to be present in both Patient 1 and Patient 2, which was inherited from their phenotypically unaffected mother. This variant was recently associated with CS and classified as pathogenic (class 5) according to the ACMG criteria (Deignan et al., 2019). The heterozygous variant p.Ala2435Val was also found to be present in both Patient 1 and Patient 2, which was inherited from their phenotypically unaffected mother. The co-segregation of p.Ala2435Val with the heterozygous p.Phe2268fs*24 in all three individuals suggested the presence of a complex VPS13B allele. Based on this, p.Ala2435Val was classified as a VUS (class 3) according to the ACMG guidelines, and current evidence suggests that it may not have a pathogenic impact. Patient 1 had the heterozygous LoF variant p.Tyr3434*, which had not been previously described. This variant appeared *de novo* in Patient 1 and was classified as pathogenic (class 5) due to the presence of a premature stop codon. The rare heterozygous v*ariant* p.Arg237Pro was found to be present in both Patient 1 and Patient 2 and was inherited from their phenotypically unaffected father. So far, this variant has been annotated in ClinVar as a VUS. Our analysis activated the ACMG criteria PM2, PP3, and PM3 for this variant. Importantly, diminished Golgi enrichment in the subcellular analysis of VPS13B p.Arg237Pro allowed for the additional activation of the PS3 term, resulting in the ACMG classification as likely pathogenic, class 4. In addition, a comparable substitution at the same amino acid position (p.Arg237His) has previously been described as a variant of uncertain significance in a compound heterozygous context, based on a trio exome analysis of a patient with CS (Liu et al., 2021). The combination of genetic and functional analyses helps to elucidate how different variants affect protein function, which will be crucial in the future for explaining phenotypic variability. Overall, genetic analysis suggested that CS in both Patients 1 and 2 was caused by the compound heterozygous VPS13B variants p.Phe2268fs*24 and p.Arg237Pro. We speculated that the *de novo* event p.Tyr3434* dominated the missense variant p.Arg237Pro in Patient 1, resulting in a complete *VPS13B* LoF and, consequently, a CS phenotype that includes neutropenia. Furthermore, we hypothesized that an incomplete LoF of *VPS13B*, due to exon-skipping splice variants, larger in-frame indels, and missense variants of uncertain significance (VUS), likely contributes to the phenotypic complexity and heterogeneity of both CS and *VPS13B*-associated ASD-like behavior (Zorn et al., 2022; Afridi et al., 2024; AbdelAleem et al., 2023). This hypothesis was supported by the altered CS phenotype observed in Patient 2 with ASD-like behavior. Together, this also suggests that establishing a phenotype–genotype correlation will improve patient management by anticipating specific clinical manifestations.

VPS13B missense variants have been rarely linked to CS due to low detection rates and limited biological evidence. A total of 4,973 coding missense variants have been reported to date, of which 109 have been found in homozygosity. The clinical significance of these variants in ClinVar is categorized as follows: 27 classified as benign/likely benign, 42 with conflicting interpretations, 28 as variants of uncertain significance (VUS), and 12 without sufficient documentation. According to data from the patient cohort, literature, and ClinVar, 42 missense variants of VPS13B have been associated with CS so far (Zorn et al., 2022). VPS13B missense variants have also been linked to autism spectrum disorders (ASDs), as noted in a previous study (Yu et al., 2013). The connection between VPS13B and ASD, particularly in patients with CS, raises questions about whether some variants may contribute to ASD-like behavior (Rafiq et al., 2015; Ionita-Laza et al., 2014; Howlin, 2001). Further exploration of this possibility could help identify more specific clinical subtypes of CS, potentially allowing for personalized care and management strategies. Notably, the majority of established CS-causing VSP13B missense variants cluster within or near the Vps13 adaptor binding (VAB) domain, which facilitates the membrane recruitment of the VPS13B C-terminus to the Golgi complex (Zorn et al., 2022; Levine, 2022). The cell biological contribution of the previously described N-terminal missense variant VPS13B p.Arg237Pro, which localizes to the highly conserved N-terminal VPS13 region (VPS13_N, also known as RBG2), to CS and the ADS-like behavior is currently unclear (Levine, 2022; Velayos-Baeza et al., 2004). The VPS13B region around the position p.Arg237 is highly conserved in vertebrates. The human VPS13_N domain has not been characterized; however, a study utilizing cryo-electron microscopy analyzed the VPS13 N-terminal regions of the fungi *Chaetomium thermophilum* and yeast *Saccharomyces cerevisiae* (Li et al., 2020). The VPS13 N-terminal region was postulated to mediate the lipid membrane attachment toward the donor lipid membrane. Mutation of critical hydrophobic amino acids into charged amino acids in the Vps13p N-terminal region of *S. cerevisiae* reduced yeast sporulation, indicating defective lipid transport (Li et al., 2020). Overall, genetic analysis of VPS13B p.Arg237Pro suggested that the VPS13_N domain is critical for VPS13B function, impacts VPS13B Golgi enrichment, and may affect the stability of the VPS13B lipid membrane complex.

In conclusion, while phenotyping and genetic testing are powerful tools for the initial identification of genetic variants, functional studies are indispensable for the accurate interpretation of missense variants. By combining these strengths, especially in the era of NGS, rigorous functional analysis will enhance our ability to diagnose accurately and consequently improve our understanding of molecular pathomechanisms. Furthermore, this study highlights the importance of integrating accurate genetic testing, comprehensive evaluation, and functional analyses to elucidate the complex phenotype–genotype correlation and the pathogenic mechanisms underlying CS. Such integration not only improves accurate diagnosis but also helps identify new therapeutic targets and improve personalized patient care. To increase understanding of CS and related neurodevelopmental disorders, future research should focus on larger cohort studies to better assess the prevalence and spectrum of VPS13B variants. Functional assays, particularly *in vitro* and *in vivo* models, are essential for validating the effects of newly identified missense variants on VSP13B function and may help uncover the molecular mechanisms driving phenotypic variability. In addition, longitudinal studies, including standardized clinical assessments and precise phenotyping of patients with CS, may assist in identifying potential genetic modifiers.

## Data Availability

The original contributions presented in the study are included in the article/supplementary material, further inquiries can be directed to the corresponding author.
